# A transgender man, a cisgender woman, and assisted reproductive technologies: a Brazilian case report

**DOI:** 10.5935/1518-0557.20200024

**Published:** 2020

**Authors:** Suely de Sousa Resende, Vitor Hugo Kussumoto, Flávia Harumi Cardoso Arima, Priscila Cristine Krul, Norma Cléa Mesquita Rodovalho, Maria Risalva de Jesus Sampaio, Mayara Muneishi Alves

**Affiliations:** 1 Dra Suely Resende – Centro de Reprodução Humana Assistida – Campo Grande, MS, Brazil

**Keywords:** transgender men, fertility, pregnancy, assisted reproduction

## Abstract

Transgender men are individuals who identify as men but were assigned female at birth. Gender-afﬁrming medications include testosterone hormone therapy, known for its diverse eﬀects throughout the body, which include endometrial atrophy and the induction of amenorrhea by suppressing ovulation, without however affecting the ovarian follicle pool. This paper reports the first case in Brazil involving a transgender man and a cisgender woman attempting to form a family. A 34-year-old transgender man and a 28-year-old woman came to our assisted reproduction service. He had been on testosterone for two years. At their initial consultation, testosterone therapy was discontinued. Controlled ovarian stimulation for the transgender man was achieved using a combination of recombinant gonadotropins FSH and LH. Pituitary blockage was performed using a GnRH antagonist protocol. Twenty follicles were aspirated and 16 oocytes were retrieved, 12 of which mature. They were inseminated with donor semen. On the fifth day of development, one high quality blastocyst was transferred to the cisgender woman, resulting in an ongoing pregnancy. Five supernumerary embryos were cryopreserved. Controlled ovarian stimulation with high quality oocytes, high quality embryos, and clinical pregnancy are possible for transgender men, even with a history of testosterone use.

## INTRODUCTION

The term transgender describes a man or woman whose gender identity is incongruent with their phenotypic sex assigned at birth (Armuand *et al*., 2017; Goldman *et al*., 2017; Hoffkling *et al*., 2017; Cheng *et al*., 2019). The true prevalence of transgenderism cannot be easily determined, since some transgender people come to terms with their gender identity later in life, while others do not openly identify as transgender due to social pressure (Irwig, 2017). Nonetheless, there is an estimated 700,000-1,000,000 transgender individuals in the United States, which account for about 0.3-0.5% of the American population (Obedin-Maliver & Makadon, 2016; Hoffkling *et al*., 2017; Cheng *et al*., 2019). Although an estimated 750,000 transgender individuals live in Brazil, no other nation murders more transgender individuals in the world (IBGE, 2018).

Some transgender individuals suffer from gender dysphoria, the distress caused by a mismatch between a person's gender identity and sex assigned at birth. Treatment for gender dysphoria may include emotional, social, hormonal therapy and surgery, which may affect the gonads and potentially the gametic pool, resulting in loss of fertility (Coleman *et al*., 2012; T'Sjoen *et al*., 2013; Condat *et al*., 2018). Transgender men identify as men but were assigned female at birth. Gender-afﬁrming surgery for these individuals leads to irreversible infertility from hysterectomy and oophorectomy. Hormonal therapy includes testosterone, which has diverse eﬀects throughout the body, aﬀecting patients both physically and psychologically. The list of effects includes endometrial atrophy, ovarian cortex and stroma hyperplasia, and induction of amenorrhea by ovulation suppression. Nevertheless, testosterone apparently does not affect the ovarian follicle pool (Coleman *et al*., 2012; T'Sjoen *et al*., 2013; Armuand *et al*., 2017; Irwig, 2017; Maxwell *et al*., 2017; Adeleye *et al*., 2019; Cheng *et al*., 2019).

Several questions around childbearing by transgender men remain unanswered, such as the optimal time to preserve reproductive potential prior to or during the initiation of gender affirming treatments (Coleman *et al*., 2012; Goldman *et al*., 2017; Adeleye *et al*., 2019). Transgender individuals are free to access assisted reproductive services to preserve gametes or reproductive tissue for future use. They may also use assisted reproductive technologies (ART) if they choose to attempt to have children. While there is some data on oocyte cryopreservation for transgender men, data on ovarian stimulation and retrieval, in vitro fertilization, and pregnancy are limited (James-Abra *et al*., 2015; Goldman *et al*., 2017; Maxwell *et al*., 2017; Adeleye *et al*., 2019).

This paper describes the case of a Brazilian couple made up by a transgender man and a cisgender woman who underwent ART at our service.

## CASE DESCRIPTION

### Couple information and medical history

A 34-year-old transgender man and a 28-year-old woman sought our private assisted reproduction service with the desire to build a family. He wanted to undergo ovarian stimulation for in vitro fertilization (IVF) and then have his eggs inseminated with donor sperm, so that the resulting embryos were transferred to his partner. He had been on 250 mg of injectable testosterone (Durateston, Aspen Pharma, Brazil) for two years and had undergone a bilateral mastectomy three months prior. He was advised to discontinue testosterone therapy at the first consultation and was prescribed folic acid supplementation. The patient was advised to wait for his menstrual cycles to return. After obtaining a general medical history from both patients, the two were ordered to undergo a thorough assessment of general health. The main findings for the cisgender woman were hypothyroidism and hyperprolactinemia.

### Controlled ovarian stimulation (COS) and egg retrieval

COS for the transgender man was achieved using a combination of recombinant-hFSH 150 IU plus recombinant-hLH 75 IU (follitropin-α and lutropin-α in a 2:1 ratio; Pergoveris; EMD Serono, São Paulo, Brazil) with recombinant-hFSH 75 IU (follitropin-α; GONAL-f; EMD Serono, São Paulo, Brazil) for the first two days. On the third day, the daily dose of recombinant-hFSH (follitropin-α; GONAL-f; EMD Serono, São Paulo, Brazil) was adjusted to 150 IU plus a fixed dose of highly purified menotropin 75 IU (human menopausal gonadotropin; hMG; Menopur, Ferring, São Paulo, Brazil). On the last two days of COS, the dose of recombinant-hFSH (follitropin-α; GONAL-f; EMD Serono, São Paulo, Brazil) was adjusted to 225 IU, with the aforementioned hMG dose. Gonadotropin administration was continued up to and including the day of human chorionic gonadotropin (hCG) administration.

 Pituitary blockage was performed using a daily dose of 1.5 mg of GnRH antagonist (Cetrotide; Asta Medica, Frankfurt, Germany), beginning when at least one follicle ≥14 mm was visualized, on the 8^th^ day of COS. 

Follicular growth was monitored using transvaginal ultrasound, beginning on the first day of gonadotropin administration. When three or more follicles attained a mean diameter of 17 mm, and serum estradiol levels were noticed (2,375 pg/mL), recombinant hCG (rhCG; Ovidrel; Merck Serono, São Paulo, Brazil) was administered to trigger final follicular maturation. The oocytes were collected 35 hours after hCG administration through transvaginal ultrasound ovum pick-up, 12 days after ovarian stimulation.

Twenty follicles were aspirated and 16 oocytes were retrieved, of which 12 were metaphase II (MII), two were metaphase I, and two were prophase I. Fertilization of 12 MII oocytes took place by intracytoplasmic sperm injection (ICSI) with donor sperm. The fertilized oocytes were cultured and resulted in eight two-pronuclear (2PN) zygotes, one 1PN zygote, and three non-fertilized (NF) oocytes.

### Embryo transfer (ET) and luteal phase support

The embryos were cultured until day 5 (D5), when one high quality blastocyst was transferred to the cisgender woman. Five supernumerary embryos were cryopreserved.

The cisgender partner had undergone cycle synchronization using 6mg/day of oral estradiol valerate (Primogyna, Schering, São Paulo, Brazil) for eight days, until an endometrial thickness of 5.9 mm was reached. The dose of estradiol was then adjusted to 8mg/day and transdermal estradiol 100 Mcg (Estradot; Novartis, São Paulo, Brazil) each 72 hours was added. Thirteen days later, on the day of ovum pick-up, her endometrial thickness was 7.6 mm. Luteal phase support was then conducted with micronized vaginal progesterone 400mg (Utrogestan - Farmoquimica, São Paulo, Brazil), with a dose adjustment to 800mg on the 14^th^ day until ET, with five days of progesterone.

Serum β-hCG reached 355.05mIU/L 11 days after ET, and an ongoing pregnancy was detected. 

### Ethical approval

The couple gave informed consent to having their case published.

## DISCUSSION

To our knowledge, this is the first Brazilian report of a transgender man in a couple to undergo COS for ART. Moreover, this case report contributes to the literature on family building involving transgender men previously on testosterone who had their oocytes retrieved and submitted to ICSI to produce a successful pregnancy. 

Studies have shown that approximately half of transgender individuals wish to have biological children, as also do cisgender individuals (Condat *et al*., 2018). The World Professional Association for Transgender Health (WPATH) Standards of Care recommends that fertility preservation options be discussed with transgender individuals with an interest in having genetically related children prior to the start of gender-affirming treatments (Coleman *et al* ., 2012; Condat *et al.* , 2018). Typically, transgender men take progestins or GnRH agonists for a short period of time in early hormonal therapy, and then move to testosterone for the rest of their lives (Irwig, 2017; Condat *et al*., 2018). Testosterone therapy is generally regarded as safe in the short term, but little is known about its long-term eﬀects. In most cases, testosterone leads produces reversible amenorrhea by suppressing ovulation, but follicles are not depleted from the ovaries. On the other hand, increased androgen levels may adversely affect follicle growth. Mature follicles are particularly affected due to their androgen-sensitive granulosa cell layer. In some circumstances, a polycystic ovarian morphology and ovarian hyperplasia have been described, (De Roo *et al* ., 2016; Irwig, 2017; Cheng *et al*., 2019).

Transgender men are required to discontinue testosterone therapy if they wish to undergo COS. Individuals dealing with the effects of testosterone cessation go through physical and mental challenges, including changes in odor and voice, fatigue, and resumption of menstruation, which may be perceived as a typically female characteristic. Bleeding was described as psychologically stressful and as a trigger to anxiety, exacerbated gender dysphoria, and self-harming behavior relapse (Armuand *et al*., 2017; Cheng *et al*., 2019).

ART and gamete cryopreservation have shaken conservative family arrangement patterns. Medical advances have extended parenting options of transgender individuals beyond adoption. Furthermore, the fight for equality and non-discrimination has led to wider recognition of Lesbian-Gay-Bisexual-TransIntersex-Questioning (LGBTIQ) rights, including in family law (Condat *et al* ., 2018). While society in general has come to accept gay and lesbian couples as potential parents, transgender individuals have long faced prejudice, including from health professionals (Goldman *et al*., 2017; Hoffkling *et al*., 2017). Nevertheless, transgender individuals have increasingly sought access to ART, and about 12% of transgender men who experienced pregnancy used reproductive technologies. However, transgender individuals and their partners have predominantly negative interactions with assisted reproduction when they access or attempt to procure the services of fertility clinics (James-Abra *et al*., 2015; Adeleye *et al*., 2019).

Our data show that an antagonist-based protocol is suitable for ovarian stimulation in this case. In our case, a transgender man had taken testosterone for two years prior to COS, with discontinuation occurring only at the initial consultation. Although lower in transgender men with a history of testosterone use, peak estradiol in this case was high (2,375pg/mL). From 20 follicles aspirated, 16 oocytes were retrieved, 12 of which mature (75%). On D5, six embryos were in culture and five were cryopreserved, with one high quality blastocyst being transferred. This may suggest that the follicular development and oocyte quality was not significantly impacted by prior testosterone therapy.

Recent studies described cases of transgender men undergoing COS for oocyte cryopreservation (Maxwell *et al*., 2017; Leung *et al*., 2018; Adeleye *et al*., 2019). Maxwell *et al*. (2017) reported the cases of three transgender men who underwent oocyte cryopreservation before starting gender-affirming hormone therapy. Two patients returned for ovum pick up and had COS performed with a low-dose leuprolide acetate protocol; both had twin pregnancies carried by their partners.

A related study from Leung *et al*. (2018) described a cohort of 22 transgender men who underwent 25 COS cycles. The authors compared their outcomes to the results attained in 75 COS cycles performed with cisgender women. The mean number of oocytes retrieved and the number of MII oocytes in the transgender group (19.7 and 14.6, respectively) were significantly higher than the numbers found in the control group (13.2 and 10.6). Peak estradiol levels and mean total gonadotropin doses used in stimulation were similar in the two groups. The mean number of fertilized oocytes of the 11 transgender men who proceeded to oocyte fertilization was not significantly different from the number seen in the control group. Recently, Leung *et al*. (2019) published more results regarding this cohort. A total of 12 embryo transfers were performed in seven patient couples, five of which were fresh embryo transfers and seven were frozen-thawed embryo transfers. The pregnancy rate was 83.3% and the live birth rate was 58.3%.

Adeleye *et al*. (2019) reported the cases of 13 transgender men who underwent COS. Seven had been on testosterone for a median length of 46 months, and had COS only six months after the discontinuation of testosterone therapy. Interestingly, a comparison between transgender men with and without a history of testosterone use found no differences in baseline follicle count, cycle length, or FSH and hMG use. Transgender men with a history of testosterone use had lower peak estradiol levels and fewer oocytes retrieved compared to transgender men without a history of testosterone use. There were no differences in estradiol levels per oocyte, MII oocyte yield, or maturity rates between the two groups. A comparison between transgender men on testosterone and cisgender women found that peak estradiol levels were lower in transgender men. This is possibly due to the latter's lower follicle counts, although there was no statistical difference in the number of oocytes and remaining cycle characteristics. However, estradiol levels per oocyte were similar between the two groups, suggesting that granulosa cell function was maintained.

Similarly to our case, three successful pregnancies were developed using the oocytes of transgender men with a history of testosterone use, although one ended in a miscarriage. Leung *et al*. (2019) reported similar peak estradiol levels between transgender men with a history of testosterone use and cisgender women, but higher gonadotropin doses were administered to the transgender men group. Retrieved and mature oocytes were also similar between the two groups, suggesting that even long periods of gender-affirming androgen therapy do not appear to have negative effects on ovarian stimulation outcomes.

## CONCLUSION

Controlled ovarian stimulation with high quality oocytes and high quality embryos leading to clinical pregnancy is a possibility for transgender men, even with a history of testosterone use. This paper describes the first Brazilian report of a couple featuring a transgender man with a history of testosterone therapy who underwent controlled ovarian stimulation, with resulting eggs submitted to intracytoplasmic sperm injection, followed by an embryo transfer that ultimately resulted in his cisgender partner having an ongoing pregnancy.
